# Advancing Clinical Insight, Medical Education, and Global Health: A Synthesis of Evidence-Based Research and Interdisciplinary Innovation

**DOI:** 10.51894/001c.160082

**Published:** 2026-04-03

**Authors:** Francis Akenami, Rana Ismail, Andrea Amalfitano

**Affiliations:** 1 College of Osteopathic Medicine – Graduate Medical Education Alliance Michigan State University, East Lansing, MI, USA

## INTRODUCTION

The *Spartan Medical Research Journal (SMRJ)* continues its mission of advancing evidence-based medicine, medical education, and interdisciplinary scholarship through the diverse contributions featured in Volume 10, Issue 3 (2025). Building upon prior work emphasizing translational and clinical impact,^1^ this issue brings together seven studies spanning graduate medical education, cardiovascular medicine, orthopedic surgery, otolaryngology workforce trends, infectious disease control, and diagnostic innovation.

Collectively, these articles highlight the evolving interface between clinical practice, educational frameworks, and global health systems, underscoring the importance of data-driven approaches to improving patient outcomes and healthcare delivery.

## INNOVATIONS IN MEDICAL EDUCATION AND TRAINING

Keyes et al. present the first national characterization of Undergraduate Research Associate Programs (RAPs) in the United States, demonstrating their growing role in supporting graduate medical education and clinical research productivity.^2^ Their findings reveal that RAPs actively engage students in diverse scholarly activities—from protocol development to manuscript preparation—while emphasizing research ethics and patient confidentiality training. This work provides a foundational framework for institutions seeking to expand structured research pipelines and cultivate early academic engagement.

Complementing this educational focus, Xu et al. evaluate a peer-to-peer case review model aimed at improving sepsis education among emergency medicine residents.^3^ Their results demonstrate significant improvements in knowledge acquisition and modest gains in diagnostic confidence following structured, asynchronous case-based learning. Together, these studies reinforce the importance of innovative, learner-centered educational strategies in addressing gaps in clinical training and quality improvement.

## EVOLVING TRENDS IN GRADUATE MEDICAL EDUCATION

Reardon et al. examine the impact of the ACGME Single Accreditation System (SAS) on osteopathic participation in otolaryngology residency programs.^4^ Their longitudinal analysis reveals a substantial increase in osteopathic match rates following the merger, without adversely affecting allopathic applicants. These findings highlight a pivotal shift toward greater equity and integration within graduate medical education, with important implications for workforce diversity and specialty access.

## CARDIOVASCULAR AND MULTISYSTEM DISEASE INTERACTIONS

Thomas et al. explore the interplay between atrial fibrillation (AF) and obstructive sleep apnea (OSA), demonstrating that the presence of OSA significantly influences clinical management strategies.^5^ Specifically, rate control predominates in patients with OSA, while rhythm control is more common in those without. These findings emphasize the need for integrated, comorbidity-informed treatment approaches in cardiovascular care.

## ADVANCES IN ORTHOPEDIC SURGICAL OUTCOMES

Sahoo et al. evaluate functional outcomes following uncemented hydroxyapatite-coated bipolar hemiarthroplasty in elderly patients with femoral neck fractures.^6^ Their prospective findings demonstrate high rates of excellent and good outcomes, with significant improvements in mobility and low complication rates. This study supports the growing adoption of biologically integrative implant technologies aimed at improving recovery in geriatric populations.

## DIAGNOSTIC INNOVATION AND MULTIDISCIPLINARY APPROACHES

Patil et al. provide a comprehensive review of advancements in the diagnostic workup of vertigo, highlighting emerging tools including clinical algorithms, risk stratification methods, and artificial intelligence applications.^7^ Despite persistent diagnostic challenges, these innovations show promise in improving accuracy and efficiency in both emergency and primary care settings. The integration of technology into diagnostic pathways reflects a broader trend toward precision and efficiency in clinical evaluation.

## GLOBAL HEALTH AND INFECTIOUS DISEASE CONTROL

Faisal et al. address critical gaps in tuberculosis (TB) preventive care in high-burden settings, identifying substantial attrition across the care cascade—from contact identification to treatment initiation.^8^ Their findings reveal that only a small proportion of eligible contacts complete preventive therapy, with major losses occurring prior to screening and diagnostic evaluation. This study underscores the urgent need for system-level interventions to strengthen TB prevention strategies and reduce missed opportunities in global health programs.

## CONCLUSION

The studies presented in this issue collectively reflect the dynamic and interconnected nature of modern healthcare, where advances in education, clinical practice, and global health systems converge to shape patient outcomes. From enhancing research training pipelines and improving diagnostic accuracy to addressing healthcare inequities and optimizing treatment strategies, these contributions exemplify the mission of SMRJ to promote impactful, interdisciplinary scholarship.

As healthcare systems continue to evolve, the integration of innovative educational models, data-driven clinical decision-making, and global health initiatives will remain essential in advancing both patient care and medical knowledge.
